# Variation of temperature increase rate in the Northern Hemisphere according to latitude, longitude and altitude: the Turkey example

**DOI:** 10.1038/s41598-024-68164-6

**Published:** 2024-08-06

**Authors:** Fatih Şevgin, Ali Öztürk

**Affiliations:** Vocational School of Technical Sciences, Muş Alparslan University, 49100 Muş, Turkey

**Keywords:** Global warming, Air temperature, Singular spectrum analysis (SSA), Least square polynomial fit (LSPF), Climate change, Civil engineering

## Abstract

Global climate change notably influences meteorological variables such as temperature, affecting regions and countries worldwide. In this study, monthly average temperature data spanning 73 years (1950–2022) were analyzed for 28 stations in the city centers across seven regions of Turkey. The station warming rates (SWR) were calculated for selected stations and the overall country using Singular Spectrum Analysis (SSA) and Least Square Polynomial Fit (LSPF) methods. The temperature trend in Turkey exhibited a decline until the late 1970s, followed by a continuous rise due to global warming. Between 1980 and 2022, the average SWR in Turkey was found to be 0.52 °C/decade. The SWR was determined to be the lowest in Antakya (0.28 °C/decade) and the highest in Erzincan (0.69 °C/decade). The relationship between SWR and latitude, longitude, altitude, and distance to Null Island (D2NI) was explored through linear regression analysis. Altitude and D2NI were found to be the most significant variables, influencing the SWR. For altitude, the correlation coefficient (R) was 0.39 with a statistically significant value (*p*) of 0.039. For D2NI, R, and *p* values were 0.39 and 0.038, respectively. Furthermore, in the multiple regression analysis involving altitude and D2NI, R and *p* values were determined to be 0.50 and 0.029, respectively. Furthermore, the collinearity analysis indicates no collinearity between altitude and D2NI, suggesting that their effects are separated in the multiple regression.

## Introduction

The human race is constantly interfering with nature, causing our planet to warm up. In particular, the ozone layer in the atmosphere is thinning due to the increase in greenhouse gases. Anthropogenic global warming is due to the absorption and back-scattering of longwave radiation by greenhouse gases, especially CFCs (chlorofluorocarbons). The heat retention feature of the atmosphere prevents the seas and oceans from freezing. The sole impact of greenhouse gases is not to prevent water bodies on Earth from freezing. In broad terms, their impact is to keep the earth’s surface and lower atmosphere warmer than they otherwise would be. Additionally, over billions of years, processes such as tectonic movements, solar flares, volcanic activities, and the elliptical orbit around the sun have actively played a role in global climate change^1^. The impact of global climate change on the entire world is evident in numerous studies and research. Particularly, the most crucial variable affected meteorologically by this change is recognized as temperature. The temperature increase rate varies from region to region, basin to basin, and country to country^2^.

The Industrial Revolution, particularly initiated in England in the 1760s, spread to almost all countries in the eighteenth and nineteenth centuries. As a consequence, the emission of greenhouse gases has brought about numerous natural disasters on our planet, primarily marked by an increase in temperature. The temperature rise is one of the most distinct features of global warming. However, beyond the global phenomenon of temperature increase, the acceleration of forest fires due to scorching temperatures, the escalation of drought and desertification, reaching hazardous levels for human life, the occurrence of flood and inundation disasters, and the experience of erosion and other natural events are also associated with the widespread elevation of temperatures worldwide^3^.

Understanding the characteristics of changes in local or regional temperature distributions as a result of global warming is a crucial aspect of comprehending the impact of alterations in extreme temperatures on living organisms^4^. Despite the annual gatherings of various countries to emphasize global climate change, a solution to this issue has yet to be collectively achieved.

According to the IPCC Third Assessment Reports, it is projected that by the year 2050, there will be a temperature increase of 6–7 °C on the southern and south-western coasts of Turkey, 4 °C in the East, and 6 °C in the West^5^. This temperature rise is attributed solely to the increase in greenhouse gases^6^. During the winter season, this increase is expected to be 2–3 °C^7^. Projections suggest that in Turkey, the Aegean, Marmara, and Mediterranean regions will experience less temperature increase but more pronounced decreases in precipitation^8^.

Mountainous and highland regions are highly sensitive to climate change due to their rich biodiversity. Small changes in temperature and precipitation can significantly impact hydrology and species, causing severe social and economic consequences. These regions act as water reservoirs, storing water in snowpacks, glaciers, and rivers, and they serve as indicators of global warming due to minimal emission impacts^9^. Shifts in temperature and precipitation patterns also threaten freshwater availability^10^, leading to severe summer drying and significant impacts on both humans and ecosystems, including reduced reservoir storage, increased wildfires, drought, and shifts in aquatic life^11^.

Moreover, the dynamics of temperature and precipitation in mountainous areas influence the radiative processes described by physical laws, such as the Stefan-Boltzmann law, which governs blackbody radiation emission. The Stefan-Boltzmann law states that a cold object requires a greater temperature increase than a hot one to emit the same additional radiation. This nonlinearity affects radiatively forced atmospheric temperature change in both horizontal and vertical directions^12^. At high altitudes, temperature variation amplification occurs due to increased diabatic processes in the mid and high troposphere caused by cloud condensation. The surface radiation balance favors latent heat over sensible heat, maintaining a ratio of 4 to 1. Variations in surface evaporation convert into heat upon condensation into cloud particles and ice crystals, thereby transferring surface radiation changes. The low-temperature environment further amplifies energy balance variations on surface temperature due to the Stefan-Boltzmann law, thereby heightening temperature variability at high altitudes^13^.

Studies in the literature have reported that certain elevation ranges experience more warming than others. This phenomenon is often referred to as elevation-dependent warming (EDW)^14^. On the contrary, some studies have not detected such a phenomenon in their analyses. Some of these studies are summarised below.

Wang et al.^15^ suggested that the warming at high-altitude stations (> 500 m above sea level) is significantly stronger than at low altitudes, by analyzing annual mean temperatures from 2781 global stations between 1961 and 2010. The data used in this study consist of 1860 stations from Global Historical Climatology Network monthly (GHCNM) mean temperature dataset, 464 stations from the National Meteorological Information Center of China (NMICC), 360 stations from Russian Meteorological Stations (RMS), 72 stations from the Historical Instrumental Climatological Surface Time Series of the Greater Alpine Region (HISTALP), and 25 stations from MeteoSwiss. In the study, it is indicated that the warming at high-elevation stations is directly proportional to temperature lapse rates along altitudinal and latitudinal gradients, by underlining that this relationship arises from the functional shape of the Stefan-Boltzmann law in both vertical and latitudinal directions. In this study, besides the high number of stations, many averages were taken to facilitate data analysis. This inevitably leads to low resolution despite the high number of stations. Data were taken from different stations in a wide geographical area around the world and annual averages of these data were used. On the other hand, since the stations are spread over a large region worldwide, the data were grouped and analyzed by averaging between the groups. The temperature trend is expressed as the slope of a linear regression. Moreover, a stepwise regression analysis is utilized to observe the relationship between variables. In a different study, Wang et al.^16^ suggested that the amplification of warming in high-elevation regions is a characteristic aspect of recent global warming. The data used in this study consists of 1861 stations from GHCNM, 499 stations from NMICC, 72 stations from HISTALP, and 25 stations from MeteoSwiss. Some preprocessing was applied before the analysis to fill in the gaps in the data and eliminate some outliers (the stations showing negative (cooling) trends or no trends). In this study, in order to fully observe the effect of altitude on the total warming rate, a three-variable function based on altitude, latitude, and longitude was constructed. By using the gradients of these three variable functions, the effects of altitude, latitude, and longitude are expressed separately. In summary, the effects of these three variables are first combined and then separability of them is observed. In a similar study, Wang et al.^17^ examined seasonal temperature trends in high-altitude regions. They reported that the warming is the strongest in winter, followed by autumn, spring, and summer. Dimri et al.^18^ examined the trends of precipitation and temperature across different elevation ranges in the Indian Himalayan region (IHR). A linear trend extracted with linear regression. They noted an increased warming with elevation during all seasons except the monsoon in the IHR. Their study included a combined analysis of EDW and the altitudinal dependence of trends in other variables to understand the potential mechanisms responsible for precipitation and temperature changes in the IHR. The study assessed the elevation dependency of the warming rate's sensitivity to moisture trends by analyzing the latitudinal distribution of the ratio between temperature trends and near-surface specific humidity trends. They found that this pattern was reflected in the trend of downwelling long-wave radiation (DLR). The increased DLR at higher elevations could be attributed to various coupled feedbacks, including moisture sensitivity and increased cloud cover.

Conversely, observational studies do not provide a definitive picture of EDW. While numerous studies suggest a positive EDW, some indicate a decrease in warming rates with elevation, and others reveal intricate warming patterns with elevation^19^, including cases where there is no significant dependence at all. For example, Pepin and Lundquist^20^ proposed that there are no global correlations between elevation and warming rates. Despite the inclusion of a relatively large number of stations in their study, they did not explain the methodology used to gather the information. Vuille and Bradley^21^ reported a trend of decreasing warming with increasing elevation. Their temperature data consisted of monthly averages from 268 stations located between 1°N and 23°S, at elevations ranging from sea level to 5000 m. These stations are part of national meteorological networks in Ecuador, Peru, Bolivia, and Chile, or were derived from the Global Historical Climatology Network. The first difference method was used to extract the annual mean temperature trend, and whether the temperature trend varied with altitude was analyzed using both ordinary least squares and the more robust least absolute residuals (LAR) regression method. Almazroui et al.^22^ investigated the impact of population, altitude, and marine temperature on the air temperature trend using data from 24 cities in Saudi Arabia. They employed an ordinary least squares regression for trend detection and observed a national temperature trend of 0.60 or 0.51 °C/decade. Additionally, they found no significant correlation between the temperature increase and both population growth and altitude variation.

The information derived from a given dataset heavily relies on the methodology employed. The efficacy of a methodology, which may encompass multiple sub-functions, hinges on its capacity to extract information from the dataset with minimal effort or preprocessing. Even if the methodology itself is complex, its applicability to diverse datasets remains crucial. Conversely, handling a sizable volume of data for processing complicates the process, necessitating data reduction through averaging. Consequently, this diminishes resolution and raises concerns about accuracy. It underscores the importance of low time complexity in the algorithms of the methodologies utilized. With algorithms featuring low time complexity and computers equipped with ample memory, data processing becomes more manageable, allowing for the extraction of reliable information from high-resolution data.

In this study, the temperature trend is extracted using SSA, recognized as a compact and robust algorithm for this purpose. SSA is a widely acknowledged for analyzing and forecasting time series data. It stands out for its incorporation of both model-free and parametric techniques, making it a versatile methodology applicable to various challenges across different domains. Particularly effective in addressing issues in time series and digital image analysis, SSA integrates principles from classical time series analysis, multivariate statistics, geometry, dynamical systems, and signal processing. Its applications range from forecasting and imputing missing values to decomposing time series into interpretable components such as slowly varying trends, oscillatory elements, and 'structureless' noise^23,24^. Importantly, SSA's core algorithms do not rely on assumptions of parametric models or stationarity, making certain versions of SSA model-free and widely applicable^25,26^.

Since the form of the trend in the data is assumed to be unknown in advance, the non-parametric nature of the SSA algorithm makes it more robust compared to the LSPF algorithm. The predetermined polynomial degree in LSPF can significantly alter the trend shape. In this study, the trend was initially extracted from the raw data using SSA. The only assumption made here is that the trend does not repeat itself within the given time interval or that its frequency is one within the given time interval. This was verified using the Fast Fourier Transform (FFT) algorithm. Conversely, in classical SSA, the trend component is determined through visual (or manual) inspection of the w-correlation matrix graph. In this study, this task is automated within the software using FFT, enabling the application of the algorithm to multiple datasets simultaneously. After extracting the trend with SSA, the portion following the upward trend was truncated, and the truncated portion was corrected using LSPF.

Here, we summarize some literature that utilizes SSA and is relatively close to the scope of this paper. Benzi et al.^27^ applied Principal Component Analysis (PCA) and SSA to analyze temperature and precipitation fields in Sardinia. SSA proved instrumental in identifying temperature field oscillations linked to planetary waves in the Northern Hemisphere. Macias et al.^28^ investigated the 'hiatus' utilizing the global surface temperature record and HadCRUT4 database, employing SSA to separate multidecadal oscillations (MDV) from secular trends (ST). Addressing missing and outlier data in land surface temperature, Ghafarian Malamiri et al.^29^ employed SSA. Marques et al.^30^ used SSA for hydrological time series analysis, including annual precipitation, monthly runoff, and hourly water temperature, initially for component extraction and subsequently for forecasting. Majumder and Kanjilal^31^ explored chaotic behavior in sea surface temperature, employing SSA alongside SVD. Yu et al.^32^ applied SSA to retrieve atmospheric temperature profiles from hyperspectral data. Jevrejeva and Moore^33^ utilized SSA to extract trends and oscillations from historical time series in the Baltic Sea. Hudson and Leatley^34^ employed SSA to delineate trends and cycles in the flowering of eucalypt species. Unnikrishnan and Jothiprakash^35^ compared SSA with the Mann–Kendall test in extracting nonlinear rainfall trends. Cristian^36^ used SSA for temperature forecasting in Romania.

This study examines the impact of global warming on the changes in air temperature increase rates in Turkey. Latitude, longitude, altitude, and distance to Null Island variables were subjected to regression analysis with temperature increase rates.

## Material and methods

In this section, we first introduce the dataset. Subsequently, the algorithms for SSA and LSPF are presented. Finally, the interpretation of the collinearity in regression analysis is discussed.

### Dataset

In this study, a total of 28 provinces from the seven regions of Turkey (Mediterranean, Marmara, Eastern Anatolia, Aegean, Central Anatolia, Black Sea, and South-eastern Anatolia) were selected, and monthly average temperature values for the years 1950–2022 were utilized. Instead of including all 81 provinces in the country, a total of 28 provinces, those with complete data (no missing value) between 1950 to 2022, were selected and included in the calculations. Monthly average temperature data for Turkey from 1950 to 2022 were obtained from the Muş Province Directorate of Meteorology. The dataset used comprises 28 provinces, spanning 12 months each over a period of 73 years, resulting in a total of 24,528 monthly average temperature values. The numerical values of altitude, latitude, and longitude, believed to influence the temperature parameter, are presented in Table 1. Each station is located in the city center. The average temperature for each city (Tmean) is obtained by taking the mean of monthly average temperature data spanning 73 years (1950–2022). According to the table, Sivas has the lowest long-term average temperature at 9.1 °C, while the highest is in Mersin at 19.3 °C.

**Table 1:** The average temperature, elevation, latitude, and longitude values for the selected provinces.

Stations	Region	Tmean (°C)	Altitude (m)	Latitude (°N)	Longitude (°E)
Antakya	Mediterranean	18.3	67	36.21	36.16
Mersin	Mediterranean	19.3	10	36.86	34.68
Bursa	Marmara	14.6	100	40.20	29.06
Tekirdağ	Marmara	14.2	37	40.98	27.51
Çanakkale	Marmara	15.2	15	40.16	26.41
Bilecik	Marmara	12.6	500	40.15	29.98
Erzincan	Eastern Anatolia	10.9	1185	39.74	39.49
Van	Eastern Anatolia	9.5	1730	38.51	43.37
İzmir	Aegean	18.0	2	38.41	27.14
Manisa	Aegean	16.9	71	38.61	27.43
Uşak	Aegean	12.6	890	38.67	29.41
Aydın	Aegean	17.8	59	37.85	27.84
Afyonkarahisar	Aegean	11.3	1034	38.76	30.54
Kütahya	Aegean	10.8	970	39.42	29.98
Kırşehir	Central Anatolia	11.6	991	39.15	34.16
Ankara	Central Anatolia	12.1	938	39.91	32.85
Kayseri	Central Anatolia	10.7	1054	38.73	35.48
Sivas	Central Anatolia	9.1	1285	39.74	37.08
Çankırı	Central Anatolia	11.4	730	40.60	33.62
Çorum	Black Sea	10.8	801	40.55	34.95
Bolu	Black Sea	10.5	725	40.74	31.61
Sinop	Black Sea	14.4	27	42.03	35.16
Samsun	Black Sea	14.6	4	41.28	36.34
Giresun	Black Sea	14.7	14	40.92	38.39
Rize	Black Sea	14.5	6	41.02	40.52
Artvin	Black Sea	12.4	345	41.18	41.82
Gaziantep	South-eastern Anatolia	15.3	850	37.06	37.38
Şanlıurfa	South-eastern Anatolia	18.6	477	37.16	38.79

**Figure 1:**
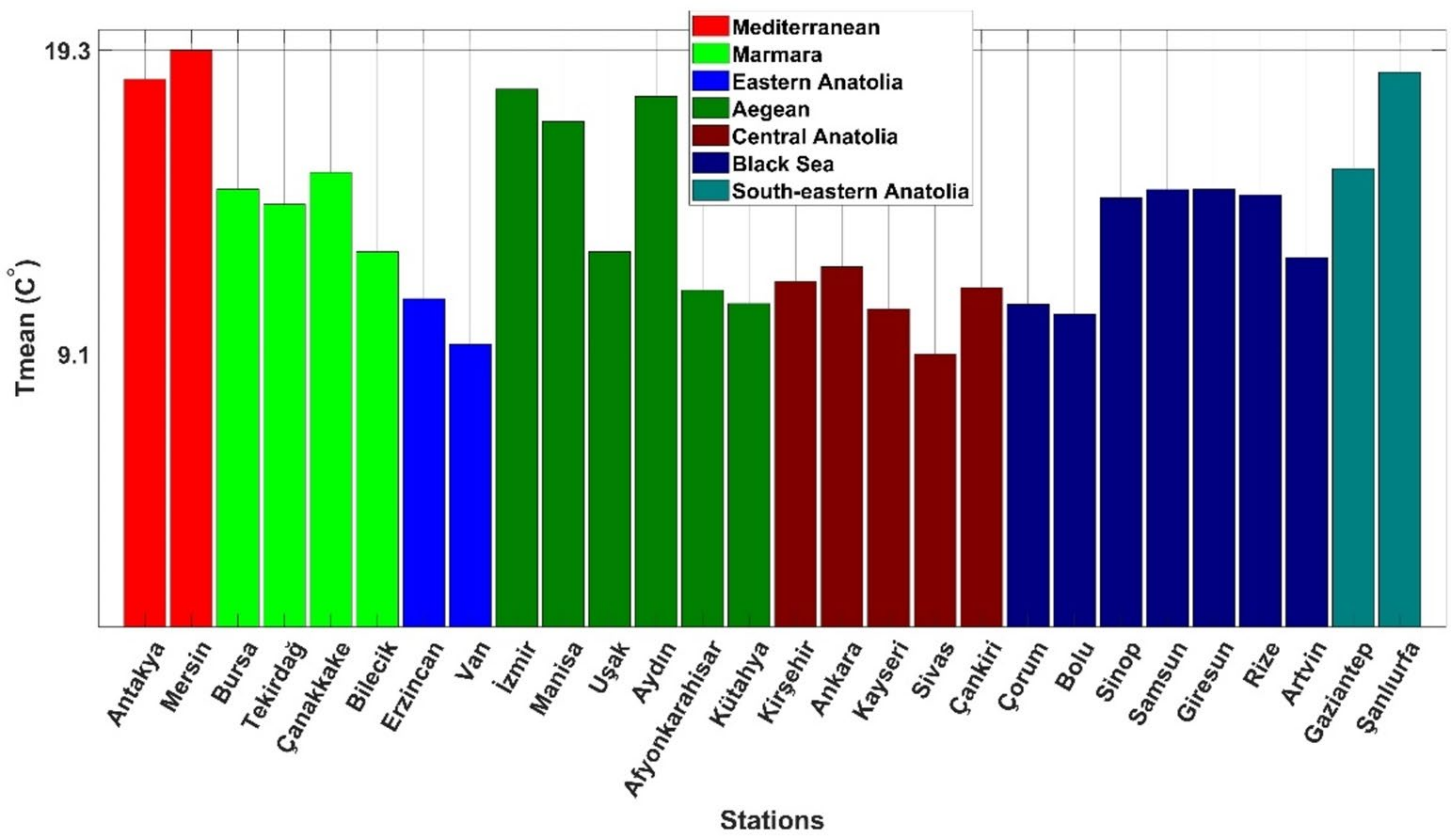
The average temperature values of the selected stations.

**Figure 2:**
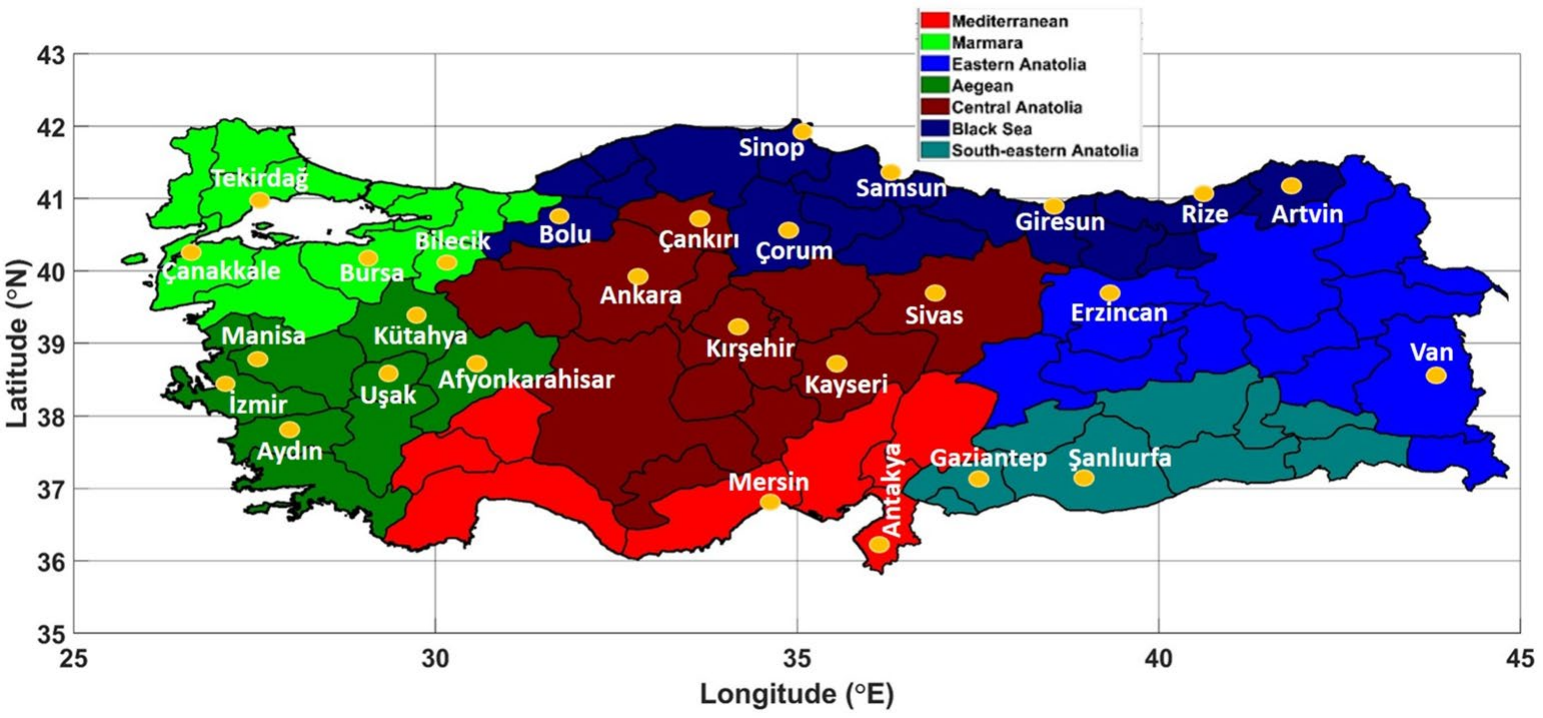
Locations of selected stations on the regional map of Turkey. (created with Mathlab R2016a).

In Fig. 1, the average temperature values for each city are given. The seven separate colored columns represent the seven regions. In Fig. 2, the locations of selected stations on the regional map of Turkey are shown. Although the altitude of Van is higher than that of Sivas, the average temperature in Sivas is lower. This clearly shows that altitude is not the only parameter affecting the temperature. For Mersin, where the highest average temperature is recorded, the values are elevation 10 m, latitude 36.86°N, and longitude 34.68°E, as shown in Table 1. According to these values, it is observed that as elevation, latitude, and longitude decrease, the average temperature increases. Regionally, the Eastern and Central Anatolia regions are found to be cooler than other regions. The main reason for this is the high elevation. However, the acceleration of global climate change in recent years has reversed this situation. In other words, in areas where elevation and latitudes have increased, the average temperature has been higher than in other areas.

**Figure 3:**
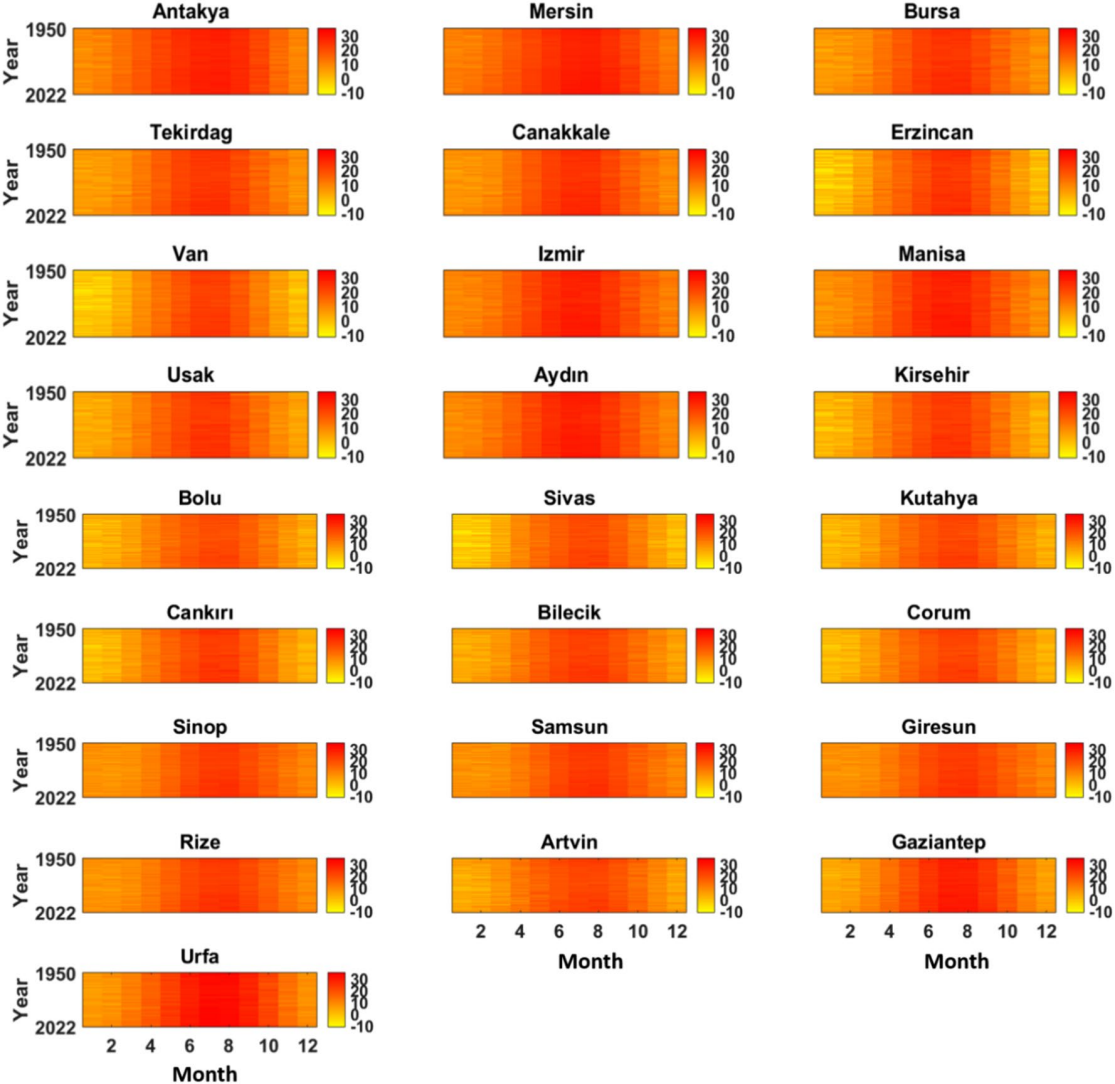
The temperature color map of the selected stations (Created with Mathlab R2016a).

When examining Fig. 3, a temperature color map for the 28 cities between the years 1950–2022 is provided. According to this map, it is observed that the monthly average temperatures throughout the year are consistently high in the Mediterranean region, particularly in the cities of Mersin and Antakya. The primary reason for this is the low elevation and proximity to the equator of these cities. In the case of Turkey, proximity to the equator has a limited impact on temperature differences among cities in the country due to its geographical location. Turkey is situated at a relatively high latitude compared to equatorial regions, meaning that seasonal variations in day length are more significant than latitudinal variations in the amount of solar radiation received. Therefore, in cities like Mersin and Antakya in the Mediterranean region, higher temperatures are primarily due to the influence of the Mediterranean Sea, and direct exposure to solar radiation for longer periods due to their southern location. In contrast, cities like Sivas in Central Anatolia experience lower temperatures due to their higher altitude, resulting in cooler climatic conditions regardless of their distance from the equator.

### Singular spectrum analysis (SSA)

The SSA algorithm is outlined in three main steps: Embedding, Singular Value Decomposition (SVD), and Separability and Reconstruction. The FFT, employed for separability in this manuscript, is also summarized.

#### Embedding

Let ${\varvec{x}}=\left({x}_{1},\cdots ,{x}_{N}\right)$ is a time series of length $N$, and consider the time series ${\varvec{x}}$ is mapped into a matrix of columns constructed with $L$-lagged vectors of size $L$ as follows;

1 $${\varvec{X}}=\left[{X}_{1} : \cdots : {X}_{K}\right]=\left(\begin{array}{ccccc}{x}_{1}& {x}_{2}& {x}_{3}& \cdots & {x}_{K}\\ {x}_{2}& {x}_{3}& {x}_{4}& \cdots & {x}_{K+1}\\ {x}_{3}& {x}_{4}& {x}_{5}& \cdots & {x}_{K+2}\\ \vdots & \vdots & \vdots & \ddots & \vdots \\ {x}_{L}& {x}_{L+1}& {x}_{L+2}& \cdots & {x}_{N}\end{array}\right)$$

In Eq. 1, the time series ${\varvec{x}}$ is mapped into a ${\varvec{X}}$ matrix of size $LxK$ and $K=N-L+1$. Such a matrix ${\varvec{X}}$ is called the trajectory (or $L$-trajectory) matrix.

#### Singular value decomposition (SVD)

Now, let us consider the SVD of ${\varvec{X}}$ as follows;

2 $${\varvec{X}}=U\Sigma {V}^{T}$$

Since ${\varvec{X}}: {\mathbb{R}}^{K}\to {\mathbb{R}}^{L}$ is a linear map (or a matrix), the column of the matrix $U$, $\left\{{u}_{1},\cdots ,{u}_{L}\right\}$ and the column of the matrix $V$, $\left\{{v}_{1},\cdots ,{v}_{K}\right\}$ are, respectively, the orthonormal bases of ${\mathbb{R}}^{L}$ and ${\mathbb{R}}^{K}$ provided by SVD and, let ${\sigma }_{1}\ge {\sigma }_{2}\ge \cdots \ge {\sigma }_{p}\ge 0$ be the corresponding singular values (where $p=min\left\{L,K\right\}$). Then, $\Sigma$ is a $LxK$ matrix $ij$ th entry is ${\sigma }_{i}$ when $i=j$ and $i\le p$, and 0 otherwise (refer to pages 143 and 146 in^37^). In that case, Eq. 2 can be re-written as follows;

3 $${\varvec{X}}={\sigma }_{1}{u}_{1}{v}_{1}^{T}+\cdots +{\sigma }_{1}{u}_{p}{v}_{p}^{T}={{\varvec{X}}}_{1}+\cdots +{{\varvec{X}}}_{p}$$

Equation 3 indicates that the trajectory matrix ${\varvec{X}}$ can be expressed as the sum of the so-called elementary matrices ${{\varvec{X}}}_{{\varvec{i}}}={\sigma }_{i}{u}_{i}{v}_{i}^{T}$. The decomposition of ${\varvec{X}}$ into elementary matrices constitutes an essential part of the SSA.

The size of the trajectory matrix could be enormous because of the time series' massive length. The classic SVD algorithms could face memory, computation time, and power constraints in such cases. Since the first few elementary components contain meaningful information, such as trend and periodicity (or seasonality), the computation of a full SVD (or ED) is unnecessary. Instead of the full SVD computation, in this work, the algorithm of the randomized SVD is utilized (refer to page 40 in^38^).

#### Separability and reconstruction

The main aim of the SSA is to separate the input time series, ${\varvec{x}}$, or its trajectory matrix, ${\varvec{X}}$, into different groups as follows;

4 $${\varvec{X}}={X}_{T}+{X}_{P}+{X}_{R}$$

In Eq. 4, ${X}_{T}$, ${X}_{P}$ and ${X}_{R}$ are respectively trend, periodicity, and residual components. Once the trajectory matrix is grouped into a set of $\left\{{X}_{T},{X}_{P},{X}_{R}\right\}$, each element of the set is reconstructed as an inverse operation of the embedding. The inverse operation of the embedding, i.e. reconstruction, is realized with the diagonal averaging. After the reconstruction with diagonal averaging Eq. 4 becomes as follows;

5 $${\varvec{x}}={x}^{T}+{x}^{P}+{x}^{R}$$

Note that, it is very computationally expensive to reconstruct each elementary matrix arising from the SVD (see Eq. 3). However, the classic separability measure, the *W*-correlation matrix, utilizes each reconstructed elementary time series. The so-called *W*-correlation between the elementary reconstructed time series ${x}^{i}$ and ${x}^{j}$ corresponding to the elementary matrices ${X}_{i}$ and ${X}_{j}$ are defined as follows;

6 $${\rho }_{w}\left({x}^{i},{x}^{j}\right)=\frac{{\langle {x}^{i},{x}^{j}\rangle }_{w}}{{\Vert {x}^{i}\Vert }_{w}{\Vert {x}^{j}\Vert }_{w}}$$

In Eq. 6, ${\langle {x}^{i},{x}^{j}\rangle }_{w}$ and ${\Vert {x}^{i}\Vert }_{w}$ are defined as follows;

7 $${\langle {x}^{i},{x}^{j}\rangle }_{w}= \sum_{k=1}^{N}{w}_{k}{x}_{k}^{i}{x}_{k}^{j} \,and\, {\Vert {x}^{i}\Vert }_{w}=\sqrt{{\langle {x}^{i},{x}^{i}\rangle }_{w}}$$

In Eq. 7, ${w}_{k}$ is the weight calculated as the number of the time series elements, ${x}_{k}$ appears in the trajectory matrix (see Eq. 1) as follows;

8 $${w}_{k}=\left\{\begin{array}{ll}i& if\quad 1\le i<L\\ L& if L\le i<K\\ N-i+1& if\quad K\le i\le N\end{array}\right.$$

The *w*-correlation’s definition in Eq. 8 considers the elementary reconstructed time series as a vector, and, utilizes the cosine formula for dot product. (https://proofwiki.org/wiki/Cosine_Formula_for_Dot_Product)

#### Fast fourier transform (FFT)

The Discrete Fourier Transform (DFT) and the FFT are mathematical algorithms used in signal processing and other fields to analyze the frequency content of a discrete signal. The DFT is a mathematical transformation that converts a sequence of complex or real numbers, representing a discrete signal in the time domain, into a sequence of complex numbers that represent the signal in the frequency domain. Given a sequence ${x}_{n}$ of length *N*, the DFT is defined by the formula;

9 $${X}_{k}=\sum_{n=0}^{N-1}{x}_{n}{e}^{-i\left(2\pi /N\right)kn}$$

In Eq. 9, *X*_*k*_ is the complex frequency component at index *k* in the frequency domain.

The straightforward computation of the DFT involves *O*(*N*^2^) operations, which can be computationally expensive for large *N*. The FFT is an algorithmic technique to compute the DFT and its inverse more efficiently than the standard DFT algorithm. It reduces the computational complexity from *O*(*N*^*2*^) to *O*(*NxlogN*). The FFT is particularly advantageous for large datasets because of its efficiency. It exploits the symmetry properties of the complex exponentials in the DFT computation. The most common FFT algorithm is the Cooley-Tukey algorithm (refer to page 63 in^38^), which recursively divides the DFT computation into smaller sub-problems until reaching base cases.

In this manuscript, FFT is implemented by using Matlab's 'fft' function.

**Figure 4:**
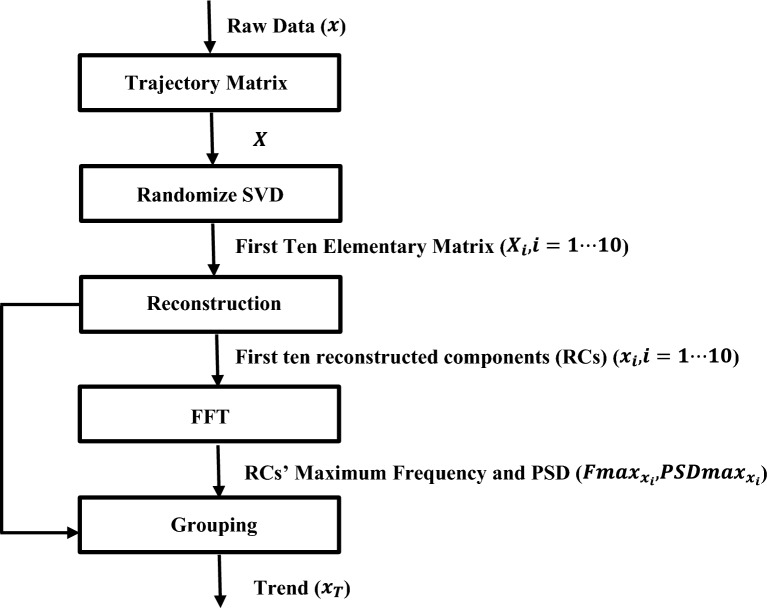
Flowchart of the SSA’s algorithm (PSD stands for power spectral density).

Figure 4 shows the outline of the overall SSA algorithm.

### Least squares polynomial fit (LSPF)

Least Squares (LS) is a mathematical methodology employed to determine the optimal curve fit, often a polynomial, for a provided dataset. The optimal curve fit is achieved by minimizing residuals, represented as the squared differences between the data points and the corresponding values on the curve.

Let ${y}_{i}$ is a discreet time series. A polynomial fit of order $n$ can be written as follows;

10 $${y}_{i}={a}_{0}+{a}_{1}{t}_{i}+{a}_{2}{t}_{i}^{2}+\cdots +{a}_{n}{t}_{i}^{n}, i=1\cdots m$$

In Eq. 10, ${y}_{i}$ represents the measurement at time (or sample) ${t}_{i}$. This system of equations can be formulated in matrix form as follows:

11 $$Y=HA$$

In Eq. 11, *Y*, *H,* and *A* are matrices with dimensions *mx1*, *mxn,* and *nx1*, respectively. The matrix *H* is referred to as a Vandermonde matrix. Generally, the number of measurements should exceed the polynomial order, ensuring that the equation system is overdetermined, i.e., *m* > *n*.

The objective of the LS is to estimate the matrix or vector of polynomial coefficients *A*, ensuring that the polynomial optimally fits the measurement samples. In other words, LS aims to determine the coefficients *A*, such that ${\Vert Y-HA\Vert }^{2}$ is minimized. Classically, this minimization can be achieved by solving Eq. 11 for *A* as follows:

12 $${H}^{T}HA={H}^{T}Y\Rightarrow A={\left({H}^{T}H\right)}^{-1}{H}^{T}Y$$

Equation 12 is referred to as the normal equation, and ${H}^{T}H$ is known as the Gramian matrix (refer to page 10 in^39^). The challenge with the Gramian matrix lies in its condition, i.e., the rank, which determines whether the column vectors of *H* are linearly independent. In large-scale problems, computing the inverse of ${H}^{T}H$ can be both computationally expensive and fraught with potential numerical instability. To circumvent issues related to ill-conditioning, it is advisable to solve the normal equation using $QR$ factorization (refer to page 124 in^40^). Furthermore, it is possible to enhance the condition number of the Vandermonde matrix *H* by substituting the ${t}_{i}, i=1\cdots m$ in Eq. 10 with equally spaced numbers in a symmetric interval, such as [− 1, 1]^41^.

## Regression analysis

We have also analyzed the collinearity between the explanatory variables that are used in multiple linear regression. Conceptually, the potential harm of collinear data is that the collinear variables do not provide much additional information beyond what is already inherent in the other variables. It therefore becomes difficult to infer the separate effects of these explanatory variables on the response variable. The formal definition of collinearity can be given as follows; the explanatory variables are collinear if one of them can be written as a linear combination of others. Therefore, collinearity is related to the ‘ill-conditioning’, i.e. rank deficiency, of the Vandermonde matrix (refer to page VII in^42^) which arises in the regression’s algorithm as a special case of LSPF. The Vandermonde matrix has already been mentioned in Section “Least squares polynomial fit (LSPF)”. Methodologically, colinearity is related to the efficiency of least squares estimation.

The condition number, which expresses the level of collinearity, is determined by the condition of the Vandermonde matrix, explaining how much it is ill-conditioned. It is determined based on the SVD. A large condition number indicates significant ill-conditioning in the Vandermonde matrix. Accordingly, a large condition number indicates strong collinearity. A condition number greater than 30 indicates strong collinearity.

In this manuscript, one-variable and multivariable regressions, along with their corresponding R and p values, are obtained using Matlab's 'fitlm' and 'regress' functions, respectively. Moreover, the collinearity test is conducted with Matlab’s built-in function ‘collintest’.

**Figure 5:**
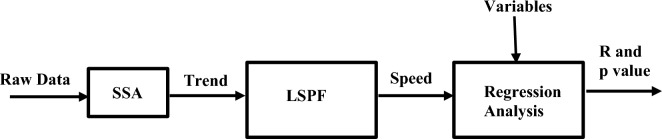
The flow diagram of the methodology applied in this study.

Figure 5 shows an outline of the flow diagram of the methodology applied in this study.

## Result

In this section, first, the computational methodology of the SWR is numerically and graphically outlined based on the mean temperature in Turkey, which is obtained by taking the mean of monthly average temperature data spanning 73 years (1950–2022) of all cities included in the study. Then, the same procedure is applied to determine the SWR of each city. Finally, the regression analysis between the SWR and the variables is presented.

### The determination of SWR using SSA and LSPF based on the mean temperature in Turkey

**Figure 6:**
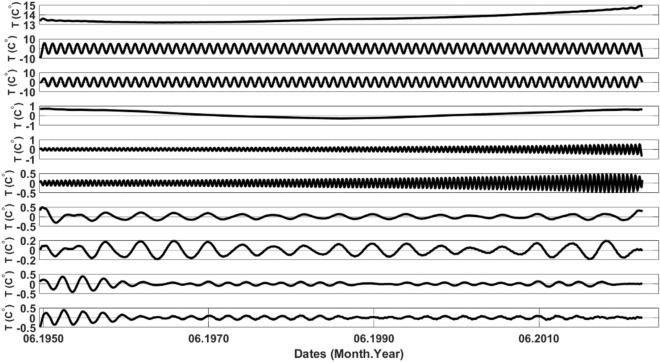
The first 10 RCs obtained through SSA are presented from top to bottom.

**Figure 7:**
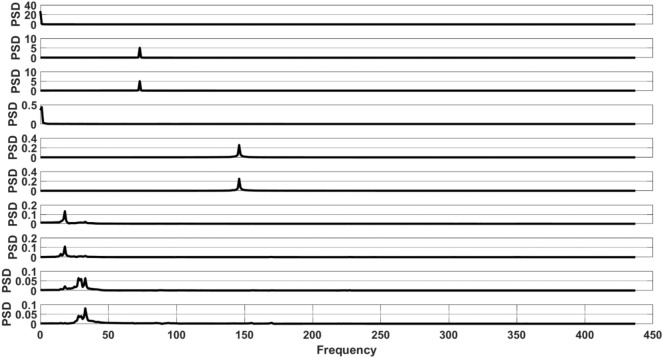
The first 10 reconstructed components of SSA are presented from top to bottom, employing the FFT.

Figure 6 illustrates the initial 10 Reconstructed Components (RCs) generated by applying the SSA algorithm with the inclusion of Turkey's mean. Subsequently, Fig. 7 depicts the Frequency versus Power Spectral Density (PSD) graphs corresponding to these components. The associated PSD_max_ and Freq_max_ values for the first 10 RCs are presented in Table 2.

**Table 2:** The maximum power spectral density (PSD_max_) and corresponding frequency (${\text{Freq}}_{\text{max}}$) values for the initial 10 RCs.

RC	PSD_max_	Freq_max_
1	27.33	0
2	5.16	73
3	5.04	73
4	0.43	1
5	0.26	146
6	0.25	146
7	0.13	18
8	0.11	18
9	0.06	28
10	0.08	33

The selection of trend components is contingent upon the criterion that the component's maximum frequency (${\text{Freq}}_{\text{max}}$) is less than or equal to 1, as outlined in Table 2. Accordingly, RC1 and RC4 are identified as trend components based on this criterion. It is noteworthy that RC2 and RC3 exhibit ${\text{Freq}}_{\text{max}}$ values of 73, aligning with the total number of years in the dataset. Consequently, these components are deemed periodic components. Components succeeding RC4 may be regarded as residuals.

**Figure 8:**
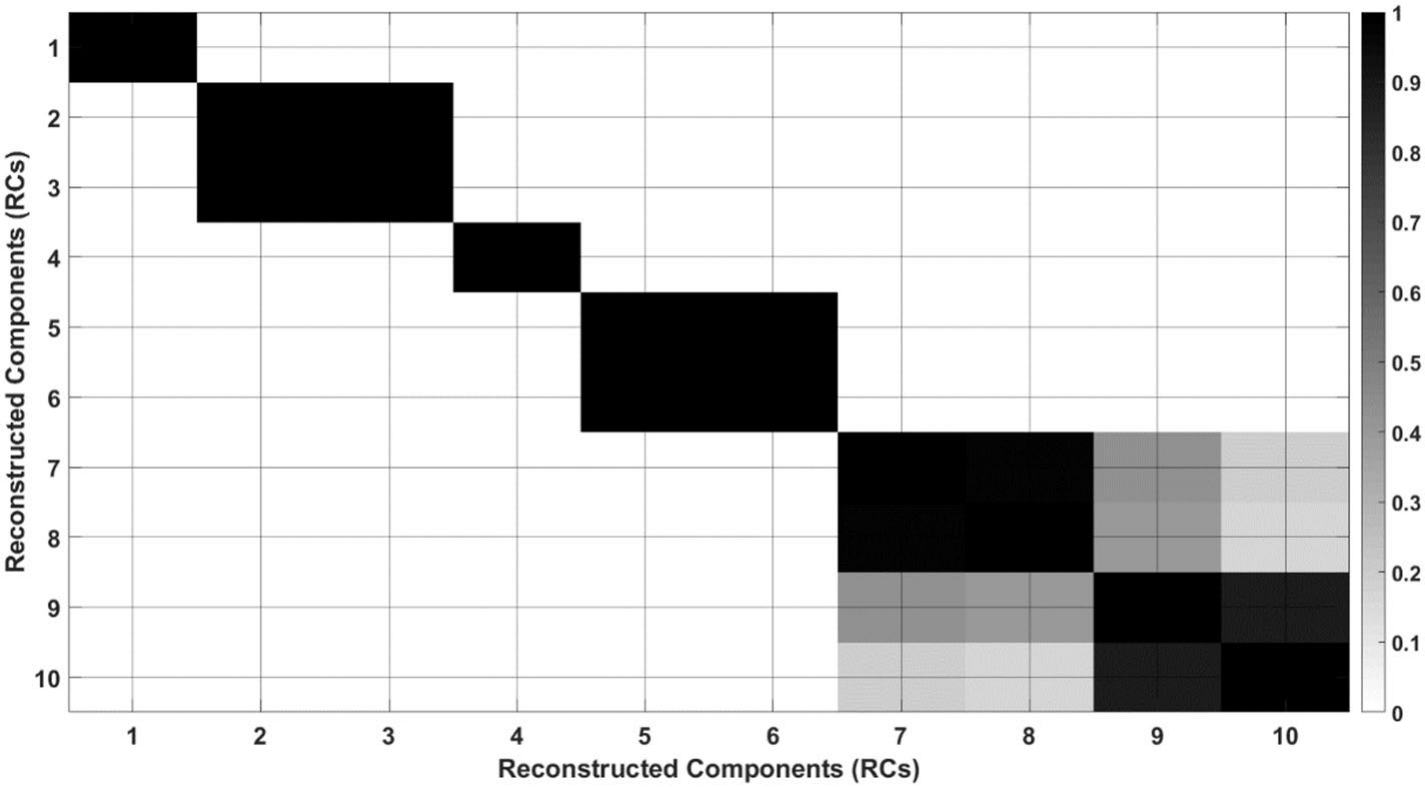
*W*-correlation matrix of the first 10 RCs.

Figure 8 illustrates the *W*-correlation matrix for the first 10 RCs. The assessment of the *W*-correlation matrix is dependent on visual inspection. Notably, this matrix lacks explicit information that precisely denotes the variability within the components. Therefore, its interpretation should be complemented by referring to Fig. 6.

Observing Fig. 8, it is noteworthy that RC1 and RC4 exhibit no correlation with any other components. Referring to Fig. 6, it becomes evident that these components possess minimal variability. Consequently, it can be inferred that RC1 and RC4 are indicative of the trend components.

Moreover, observing Fig. 8, a correlation is evident between RC2 and RC3, as well as RC5 and RC6. Nevertheless, in Fig. 6, the variability (or frequency) in RC5 and RC6 surpasses that in RC2 and RC3. Consequently, RC2 and RC3 can be identified as periodic components. The critical question arises: should RC5 and RC6 also be categorized as periodic components? Examining their **PSD**_**max**_ values in Table 2 reveals that these values are markedly smaller than those of RC2 and RC3.

In summary, the discussion highlights that separation using the FFT is more straightforward compared to the *W*-correlation matrix. Unlike the *W*-correlation matrix, FFT does not necessitate visual inspection and allows for automated separation based on a predefined threshold.

**Figure 9:**
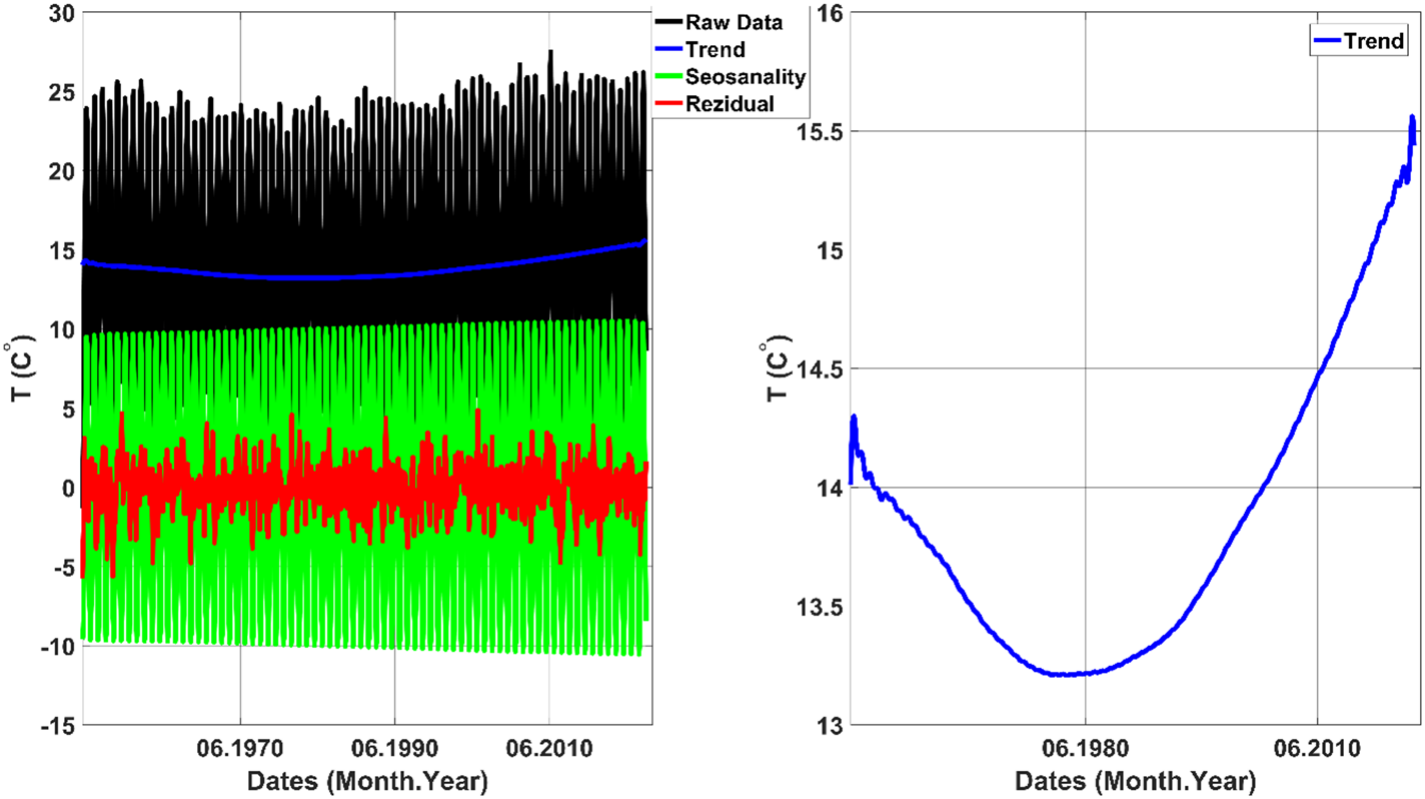
SSA of the temperature data belongs to Turkey's mean. **Left figure:** Raw data, trend, seasonality, and residual components. **Right figure:** Only trend component.

**Figure 10:**
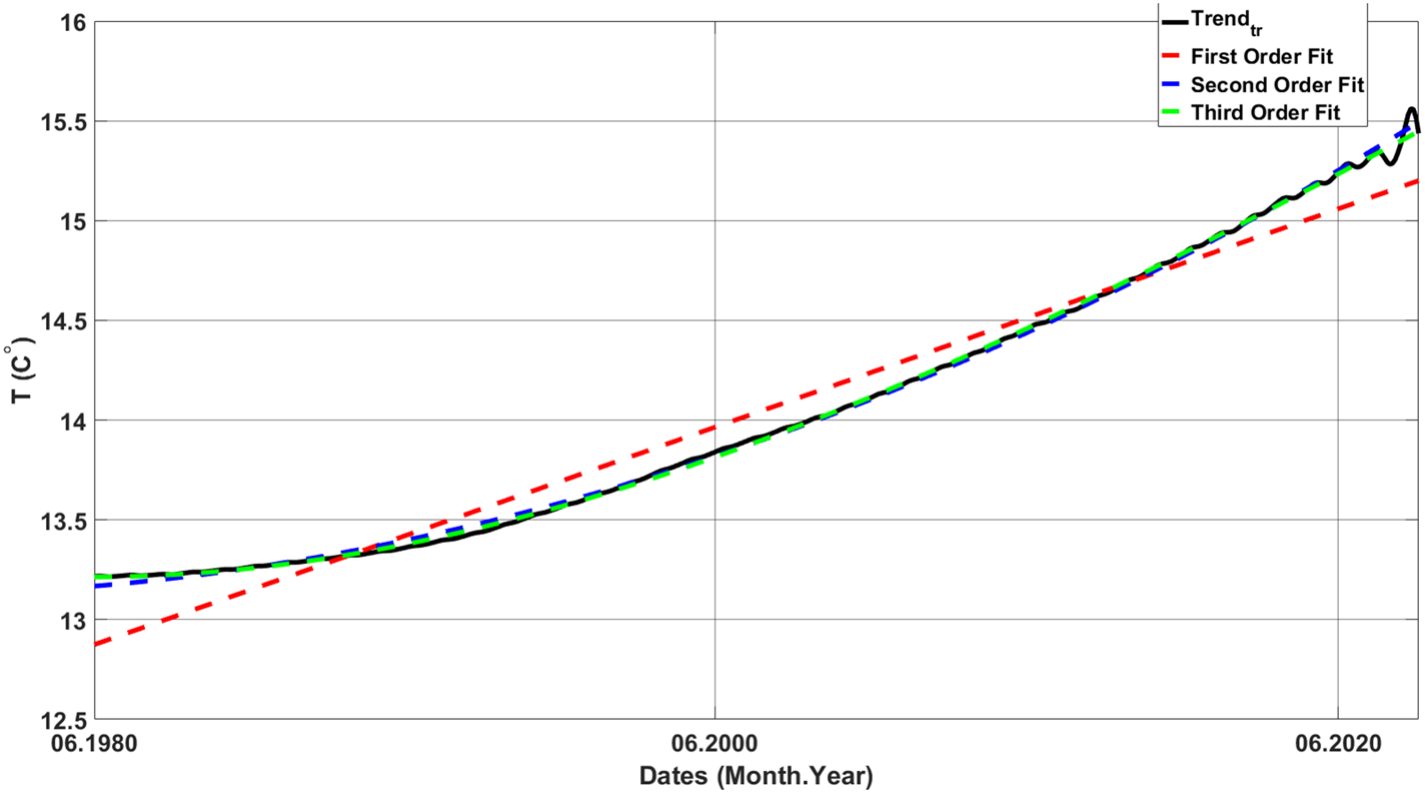
LSPF to the truncated trend (Trend_tr_).

Figure 9 depicts the outcome of the SSA analysis. It is noteworthy that the upward trend in temperature becomes evident from 1980 onwards. The trend is truncated after this specific year to calculate the SWR. Subsequently, to ensure accurate and robust SWR calculation, a polynomial fit is applied to the truncated trend (Trend_tr_). Figure 10 presents the first, second, and third-order polynomial fits, with the third-order polynomial emerging as the best fit for the data.

The SWR is determined by computing the difference between the last and first values of the third-order polynomial fit applied to the truncated trend, as outlined below:

13 $$SWR=120x\frac{PF3{|}_{last}-PF3{|}_{first}}{n}$$

In Eq. 13, *PF3* denotes the third-order polynomial fit to the truncated trend, and *n* denotes the number of months (the data length) in the truncated trend. In order to obtain speed in ‘°C/decade’ unit, the ratio is multiplied by 120. Accordingly, Turkey's mean SWR is 0.52 °C/decade.

### The SWR for each city

**Figure 11:**
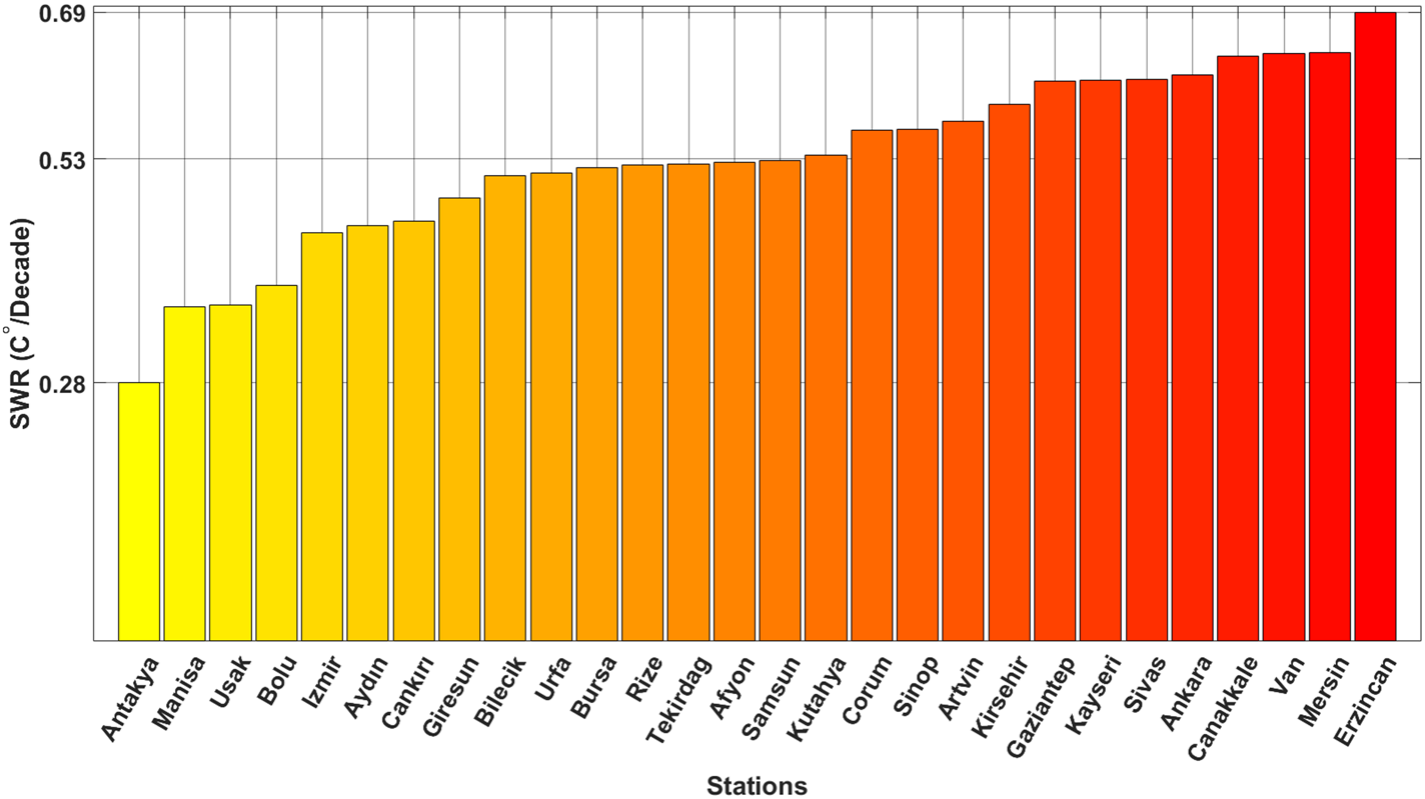
The station warming rate (SWR) of each station.

Figure 11 illustrates the SWR of each station, ordered from minimum to maximum values. Notably, Antakya exhibits the minimum SWR value (0.28 °C/decade), whereas Erzincan displays the maximum SWR value (0.69 °C/decade). Additionally, the SWR in Rize, Tekirdağ, and Afyon are relatively moderate and almost equal compared to other stations.

All stations with complete monthly data from 1950 to 2022 are included in the study. A minimum of 2 stations from each of the seven regions are included. The Mediterranean, Eastern Anatolia, and Southeastern Anatolia regions have the fewest number of stations included in the study, while the Black Sea region has the most stations. However, upon evaluating Figs. 2 and 11 together, it is observed that not only stations within the same region but also those in different regions exhibit different SWR values. The distribution of SWR values among stations in all sub-regions is not uniform. For instance, the SWR values of Mersin and Antakya cities in the Mediterranean region differ significantly from each other.

### Regression analysis

**Figure 12:**
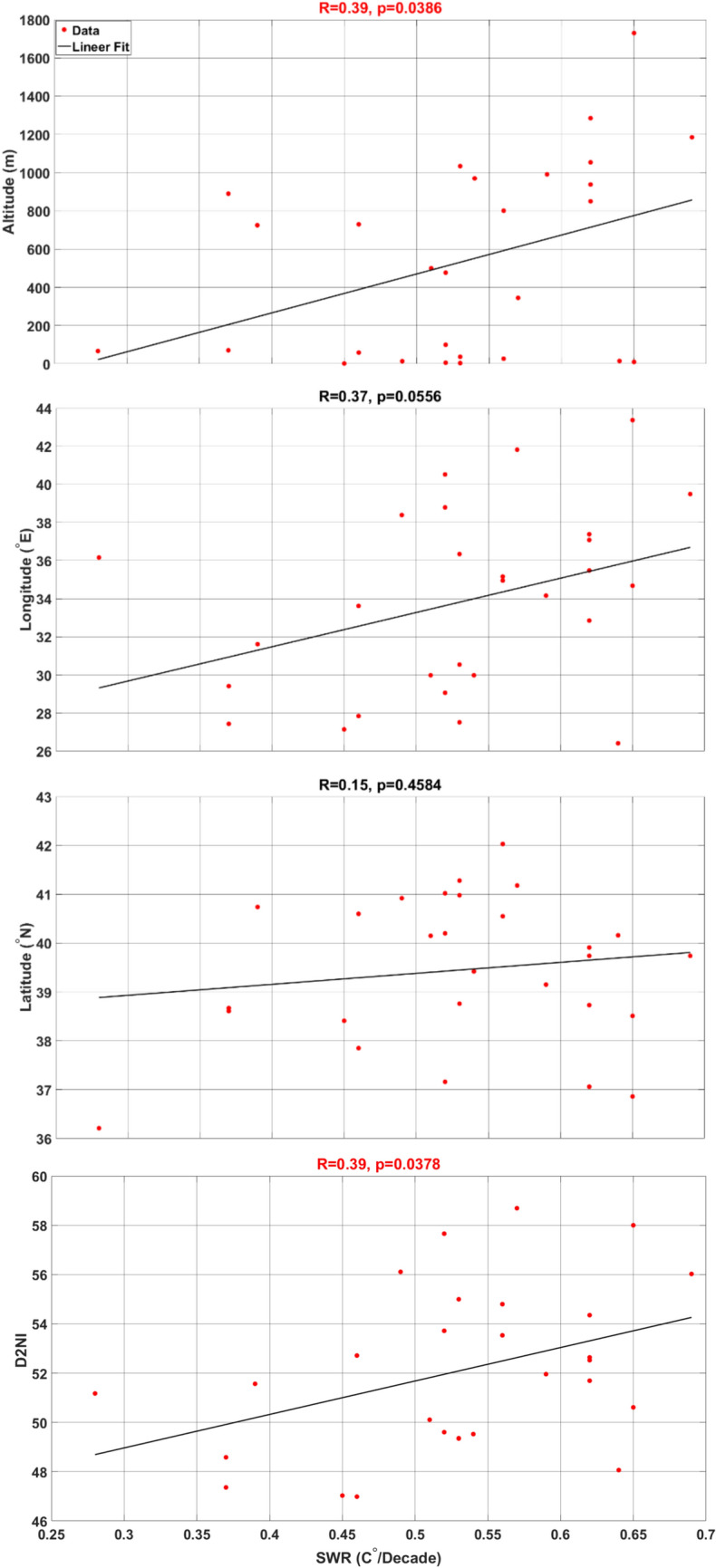
Linear regression between SWR and variables.

In Fig. 12, a one-dimensional linear regression analysis was performed to examine the relationship between the SWR and geographical factors, including latitude, longitude, altitude, and the distance to Null Island (*D2NI*). Null Island, located at the point where the prime meridian and the equator intersect (0° N, 0° E), serves as a reference point for the analysis. The *D2NI* for each city was calculated as follows;

14 $$D2NI=\sqrt{{longitude}^{2}+{latitude}^{2}}$$

In Eq. 14, D2NI is not a linear combination of longitude and latitude. Therefore, it is obvious that the collinearity is not an issue for this variable. Morever, longitude and latitude variables are linearly independent.

According to the analysis, altitude and D2NI emerge as the most significant factors influencing the SWR. The correlation coefficient of 0.39 highlights a stronger impact of altitude and D2NI compared to other variables. Furthermore, the small p-values for altitude (0.039) and D2NI (0.038), both less than 0.05, signify their statistical significance. In contrast, latitude exhibits the least impact, with a correlation value of 0.15 (*p* = 0.458), suggesting a relatively minimal effect on the SWR, rendering it statistically non-significant. Furthermore, longitude is observed to be statistically non-significant (*p* = 0.056), but its correlation relationship (R = 0.37) is found to be close to that of altitude and D2NI.

**Figure 13:**
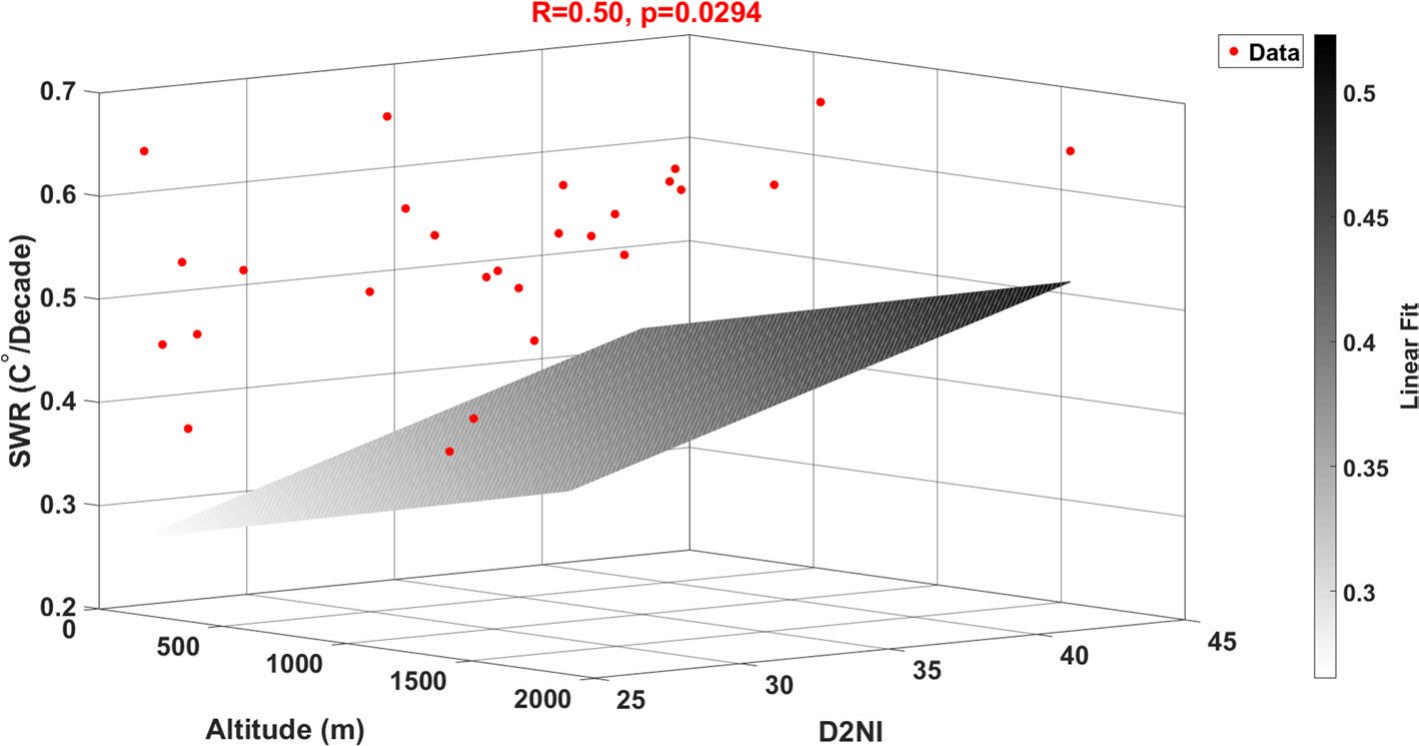
Multiple linear regression between altitude, D2NI, and SWR.

Figure 13 presents a multiple regression analysis conducted with altitude and D2NI. By this analysis, the correlation value of 0.5 and a *p*-value of 0.029 indicate that the relationship between the temperature increase rate and these two variables is statistically strong and significant.

Since altitude and D2NI are used in multiple regression (two-dimensional regression), a collinearity analysis is required for these two parameters. For the collinearity analysis design matrix is selected as $X=[{x}_{1} {x}_{2}]$ where ${x}_{1}$ and ${x}_{2}$ denote respectively altitude and D2NI column vectors of that size are $28x1$.

In Table 3, a column of sValue shows a vector of singular values of the scaled design matrix X, in descending order. The column of condIdx shows a vector of condition indices sValue(1)/sValue(j), j = 1,2, in ascending order. Large indices identify high collinearity among the variables in X. The column of altitude and D2NI shows an array of variance-decomposition proportions. A large proportion, combined with a large condIdx indicates a high collinearity. Since the second value of the condIdx column (2.59) of Table 3 is substantially smaller than the threshold value of 30, there is no collinearity between altitude and D2NI. Therefore, it can be said that they do not impose a mixture effect on the SWR. In other words, their impact on SWR are well separated.

**Table 3:** Colinearity analysis between altitude and D2NI.

sValue	condIdx	Altitude	D2NI
1.32	1	0.13	0.13
0.51	2.59	0.87	0.87

## Conclusion and discussion

In this study, data from 28 provinces across seven regions of Turkey were analyzed to assess the temperature increase rates within these provinces. Monthly average temperature values spanning 73 years, from 1950 to 2022, were used in the analysis.

The SWR for each station and the average for Turkey were calculated using the SSA and LSPF methods.

In the literature, trend extraction from temperature data is generally performed with LSPF. In this study, SSA, which is a more robust algorithm, is used in this task. Although there are similar studies in the literature, our study differs in this respect. Demonstrating the relationship between SWR and elevation with a different dataset and a different methodology strengthens the existing hypotheses in this direction.

In the analysis of this work, the temperature trend in Turkey, attributed to global warming, showed a decline until the late 1970s, followed by a continuous rise after the 1980s. Natural variability in climate systems, such as the Atlantic Multidecadal Oscillation (AMO), the North Atlantic Oscillation (NAO), and solar cycles, can significantly impact regional climates, including Turkey.

During the cool phase of the AMO (1960s–1970s), Turkey experienced relatively stable or cooling temperatures. The transition to the warm phase in the late 1970s and early 1980s coincides with the observed increase in temperatures^43^. The NAO, a weather phenomenon in the North Atlantic Ocean, influences the climate of Europe and the Mediterranean. Positive and negative phases of the NAO can lead to variations in temperature and precipitation patterns. NAO's positive phases generally result in warmer and wetter winters in Turkey. The variability of the NAO index during the studied period could contribute to the temperature trends^44^.

Changes in solar radiation and its impact on Earth's climate have contributed to the natural variability observed in temperature trends. Solar cycles can influence short-term climate variability^45^. Additionally, increased aerosol emissions from industrial activities and volcanic eruptions during the mid-twentieth century contributed to global dimming, reflecting sunlight and temporarily cooling the Earth's surface^46^.

The temperature trends in Turkey, characterized by a decline until the late 1970s followed by a continuous rise after the 1980s, can be attributed to a combination of natural climate variability and anthropogenic factors. The shift from a relatively stable or cooling trend to a warming trend aligns with changes in the AMO and NAO, solar cycles, global increases in greenhouse gas emissions, and regional influences such as urbanization. This multifaceted approach helps explain the observed temperature patterns and provides a comprehensive understanding of the underlying causes.

For the average temperature in Turkey, the SWR between 1980 and 2022 was found to be 0.52 °C/decade. When examining individual stations, the lowest SWR value was observed in Antakya (0.28 °C/decade), while the highest SWR value occurred in Erzincan (0.69 °C/decade). This pattern can be explained by the shift of global warming from the Mediterranean region towards the inland areas of Turkey and the transition from low to high altitudes.

The relationship between the SWR and latitude, longitude, elevation, and D2NI was explored through linear regression analysis. It was observed that altitude and D2NI were the most significant variables influencing the SWR.

Additionally, the latitude and longitude variables were found to be statistically non-significant in relation to the SWR. The fact that the p-value indicating the level of statistical significance is less than 0.05 indicates that the relationship between the SWR and not only altitude (*p* = 0.039) but also D2NI (*p* = 0.038) parameters can be extended to a larger sample. Moreover, collinearity analysis between altitude and D2NI showed that there is no collinearity (condIdx = 2.6 < 30) between these two parameters. This shows that the effect of these two variables on SWR can be distinguished. In the multiple regression in which altitude and D2NI were used as independent variables and SWR as the dependent variable, it was observed that both the R-value became stronger (R = 0.5) and the p-value became smaller (*p* = 0.029) and gained more statistical significance. This means that the combination of altitude and D2NI provides a more accurate and reliable explanation of the variation in SWR compared to models that do not include these variables.

The analysis and findings indicate that altitude and distance to Null Island (D2NI) are significant predictors of the SWR. The absence of collinearity between these variables allows for a clear differentiation of their individual effects. The multiple regression model incorporating these variables shows a stronger and more statistically significant relationship with SWR, underscoring their importance in climate studies.

It is challenging to find direct references in the literature on the use of the D2NI parameter in climate change studies. However, to address the specific finding that in Turkey, the station warming rate increases with D2NI rather than latitude, we should explore the unique climatic, geographical, and anthropogenic factors contributing to this pattern.

Turkey's diverse topography, including mountains, plateaus, and coastal regions, significantly influences local climate patterns. Elevation and terrain variability might impact warming rates more substantially than latitude alone. Additionally, Turkey's location between the Mediterranean and continental climate zones results in complex climatic interactions. The distance from the equator and proximity to large water bodies create unique microclimates with varying warming rates. The Mediterranean climate, characterized by hot, dry summers and mild, wet winters, contributes to distinct warming trends in regions farther from the equator^47^.

Furthermore, the North Atlantic Oscillation (NAO) significantly influences weather patterns in Turkey. Positive and negative phases of the NAO affect temperature and precipitation, potentially impacting warming rates differently across the country^48^. The finding that the SWR in Turkey increases with D2NI rather than latitude can be explained by considering the unique geographical, climatic, and anthropogenic factors at play. Turkey’s diverse topography, the influence of the Mediterranean climate, and regional effects of the NAO may contribute to this pattern. However, the main reason may be that Turkey is located in a narrow latitudinal range and the D2NI parameter’s range, the diagonal extending from southwest to northeast, has a much wider range than the latitude parameter.

In particular, our findings suggest that the Stefan-Boltzmann law may serve as an important mechanism explaining an increase in the SWR increase at high altitudes and with increasing distance between the stations and the NULL island.

## Data Availability

The data and Matlab code for the algorithm that generates each figure related to the data in the manuscript are available here: https://github.com/ozturk-ali/TurkeyWarming.
